# {6,6′-Dimeth­oxy-2,2′-[4,5-dimethyl-*o*-phenyl­enebis(nitrilo­methyl­idyne)]diphenolato}nickel(II)

**DOI:** 10.1107/S1600536810035890

**Published:** 2010-09-11

**Authors:** Hadi Kargar, Reza Kia, Muhammad Nawaz Tahir, Atefeh Sahraei

**Affiliations:** a Department of Chemistry, School of Science, Payame Noor University (PNU), Ardakan, Yazd, Iran; b Department of Chemistry, Science and Research Branch, Islamic Azad University, Tehran, Iran; c X-ray Crystallography Lab., Plasma Physics Research Center, Science and Research Branch, Islamic Azad University, Tehran, Iran; d Department of Physics, University of Sargodha, Punjab, Pakistan

## Abstract

In the title Schiff base complex, [Ni(C_24_H_22_N_2_O_4_)], the Ni^II^ atom has a slightly distorted square-planar coordination environment. The dihedral angles between the central benzene ring and the two outer rings are 7.62 (16) and 9.78 (17)°. The crystal structure is stabilized by inter­molecular C—H⋯O hydrogen bonds and π–π inter­actions with a centroid–centroid distance of 3.8218 (19) Å.

## Related literature

For background to Schiff base–metal complexes, see: Granovski *et al.* (1993 ▶); Blower *et al.* (1998 ▶); Elmali *et al.* (2000 ▶). For standard values of bond lengths, see: Allen *et al.* (1987 ▶).
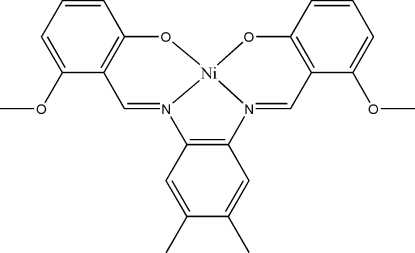


## Experimental

### 

#### Crystal data

[Ni(C_24_H_22_N_2_O_4_)]
*M* *_r_* = 461.15
Monoclinic, 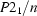

*a* = 12.8057 (6) Å
*b* = 12.6514 (5) Å
*c* = 13.0263 (6) Å
β = 101.730 (2)°
*V* = 2066.32 (16) Å^3^
*Z* = 4
Mo *K*α radiation
μ = 0.97 mm^−1^
*T* = 296 K
0.24 × 0.14 × 0.08 mm

#### Data collection

Bruker SMART APEXII CCD area-detector diffractometer
Absorption correction: multi-scan (*SADABS*; Bruker, 2005 ▶) *T*_min_ = 0.800, *T*_max_ = 0.926
36150 measured reflections
5146 independent reflections
3179 reflections with *I* > 2σ(*I*)
*R*_int_ = 0.068

#### Refinement

*R*[*F* ^2^ > 2σ(*F* ^2^)] = 0.047
*wR*(*F* ^2^) = 0.124
*S* = 1.03
5146 reflections
284 parameters
H-atom parameters constrained
Δρ_max_ = 0.48 e Å^−3^
Δρ_min_ = −0.34 e Å^−3^

### 

Data collection: *APEX2* (Bruker, 2005 ▶); cell refinement: *SAINT* (Bruker, 2005 ▶); data reduction: *SAINT*; program(s) used to solve structure: *SHELXS97* (Sheldrick, 2008 ▶); program(s) used to refine structure: *SHELXL97* (Sheldrick, 2008 ▶); molecular graphics: *SHELXTL* (Sheldrick, 2008 ▶); software used to prepare material for publication: *SHELXTL* and *PLATON* (Spek, 2009 ▶).

## Supplementary Material

Crystal structure: contains datablocks global, I. DOI: 10.1107/S1600536810035890/su2210sup1.cif

Structure factors: contains datablocks I. DOI: 10.1107/S1600536810035890/su2210Isup2.hkl

Additional supplementary materials:  crystallographic information; 3D view; checkCIF report

## Figures and Tables

**Table 1:** Hydrogen-bond geometry (Å, °)

*D*—H⋯*A*	*D*—H	H⋯*A*	*D*⋯*A*	*D*—H⋯*A*
C7—H7*C*⋯O3^i^	0.96	2.51	3.424 (5)	158
C21—H21⋯O2^ii^	0.93	2.52	3.340 (4)	147

Symmetry codes: (i) 
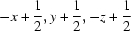
; (ii) 
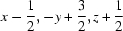
.

**Fig. 1.**
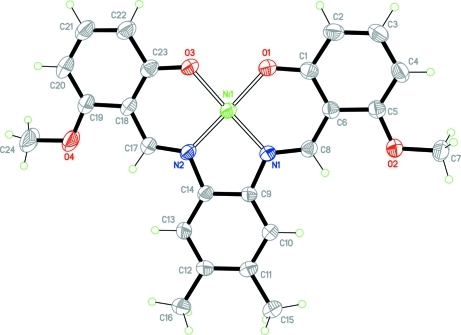
The molecular structure of the title molecule, showing 40% probability displacement ellipsoids and the atom numbering scheme.

**Fig. 2.**
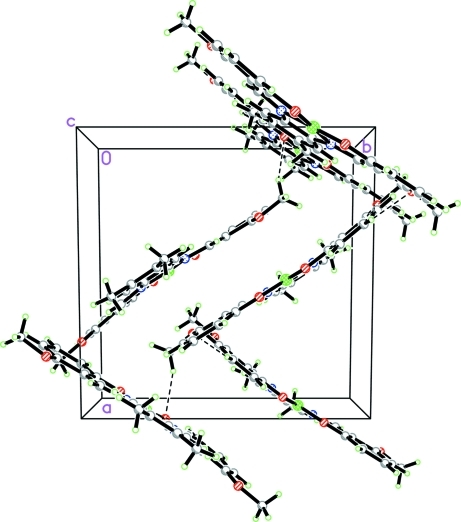
The crystal packing diagram of the title compound viewed down the c-axis showing intermolecular interactions as dashed lines.

## supplementary crystallographic information

### Comment

Schiff base complexes are one of the most important stereochemical models in transition metal coordination chemistry, with ease of preparation and structural variations (Granovski *et al.*, 1993). Metal derivatives of the Schiff bases have been studied extensively, and nickel(II) and copper(II) complexes play a major role in both synthetic and structurel research (Elmali *et al.*, 2000; Blower *et al.*, 1998).

The molecular structure of the title molecule is illustrated in Fig. 1. The bond lengths (Allen *et al.*, 1987) and angles are within normal ranges. The geometry around the Ni^II^ atom is square-planar being coordinated by the N_2_O_2_ donor atoms of the tetradenate Schiff base ligand. The dihedral angle between the mean planes of the central aromatic ring (C9-C14) with the two outer rings (C1-C6 and C18-C23) are 7.62 (16) and 9.78 (17)°, respectively.

The crystal structure is stabilized by intermolecular C—H···O hydrogen bonds (Table 1) and π–π interactions [Cg1···Cg2^i^ = 3.8218 (19)Å; Cg1 and Cg2 are the centroids of the C1-C6 and C9-C14 rings, respectively; symmetry code (i) -x, 2-y, -z].

### Experimental

The title compound was synthesized by adding bis(6-methoxysalicylidene)-4,5-dimethyl phenylenediamine (2 mmol) to a solution of NiCl_2_^.^6H_2_O (2 mmol) in ethanol (30 ml). The mixture was refluxed with stirring for 30 min. The resultant red solution was filtered. Dark-red plate-like single crystals of the title compound, suitable for *X*-ray structure analysis, were obtained by slow evaporation at RT of a solution in ethanol over a period of several days.

### Refinement

All the H-atoms were positioned geometrically and included in a riding model approximation: C—H = 0.93 and 0.96 Å for CH and CH_3_ H-atoms, respectively, with U_iso_(H) = k × U_eq_ (C), where k = 1.5 for methyl H-atoms and k = 1.2 for all other H-atoms.

### Crystal data

**Table:**

[Ni(C_24_H_22_N_2_O_4_)]	*F*(000) = 960
*M**_r_* = 461.15	*D*_x_ = 1.482 Mg m^−^^3^
Monoclinic, *P*2_1_/*n*	Mo *K*α radiation, λ = 0.71073 Å
Hall symbol: -P 2yn	Cell parameters from 2525 reflections
*a* = 12.8057 (6) Å	θ = 2.5–29.5°
*b* = 12.6514 (5) Å	µ = 0.97 mm^−^^1^
*c* = 13.0263 (6) Å	*T* = 296 K
β = 101.730 (2)°	Plate, red
*V* = 2066.32 (16) Å^3^	0.24 × 0.14 × 0.08 mm
*Z* = 4	

### Data collection

**Table:**

Bruker SMART APEXII CCD area-detector diffractometer	5146 independent reflections
Radiation source: fine-focus sealed tube	3179 reflections with *I* > 2σ(*I*)
graphite	*R*_int_ = 0.068
φ and ω scans	θ_max_ = 28.4°, θ_min_ = 2.0°
Absorption correction: multi-scan (*SADABS*; Bruker, 2005)	*h* = −17→17
*T*_min_ = 0.800, *T*_max_ = 0.926	*k* = −16→16
36150 measured reflections	*l* = −17→17

### Refinement

**Table:**

Refinement on *F*^2^	Primary atom site location: structure-invariant direct methods
Least-squares matrix: full	Secondary atom site location: difference Fourier map
*R*[*F*^2^ > 2σ(*F*^2^)] = 0.047	Hydrogen site location: inferred from neighbouring sites
*wR*(*F*^2^) = 0.124	H-atom parameters constrained
*S* = 1.03	*w* = 1/[σ^2^(*F*_o_^2^) + (0.0484*P*)^2^ + 1.1318*P*] where *P* = (*F*_o_^2^ + 2*F*_c_^2^)/3
5146 reflections	(Δ/σ)_max_ < 0.001
284 parameters	Δρ_max_ = 0.48 e Å^−^^3^
0 restraints	Δρ_min_ = −0.34 e Å^−^^3^

### Special details

**Table:**

Geometry. All esds (except the esd in the dihedral angle between two l.s. planes) are estimated using the full covariance matrix. The cell esds are taken into account individually in the estimation of esds in distances, angles and torsion angles; correlations between esds in cell parameters are only used when they are defined by crystal symmetry. An approximate (isotropic) treatment of cell esds is used for estimating esds involving l.s. planes.
Refinement. Refinement of F^2^ against ALL reflections. The weighted R-factor wR and goodness of fit S are based on F^2^, conventional R-factors R are based on F, with F set to zero for negative F^2^. The threshold expression of F^2^ > 2sigma(F^2^) is used only for calculating R-factors(gt) etc. and is not relevant to the choice of reflections for refinement. R-factors based on F^2^ are statistically about twice as large as those based on F, and R- factors based on ALL data will be even larger.

### Fractional atomic coordinates and isotropic or equivalent isotropic displacement parameters (Å^2^)

**Table:**

	*x*	*y*	*z*	*U*_iso_*/*U*_eq_	
Ni1	0.01614 (3)	0.78355 (3)	0.17262 (3)	0.03619 (14)	
O1	0.07749 (19)	0.88142 (16)	0.27017 (17)	0.0481 (6)	
O2	0.21622 (19)	1.12212 (16)	0.05147 (18)	0.0483 (6)	
O3	−0.02481 (18)	0.72048 (16)	0.28477 (17)	0.0453 (5)	
O4	−0.2538 (2)	0.4558 (2)	0.1089 (2)	0.0768 (9)	
N1	0.06210 (19)	0.84778 (18)	0.06261 (19)	0.0346 (6)	
N2	−0.0465 (2)	0.68259 (19)	0.07723 (19)	0.0364 (6)	
C1	0.1314 (3)	0.9655 (2)	0.2565 (3)	0.0410 (7)	
C2	0.1766 (3)	1.0258 (3)	0.3455 (3)	0.0506 (9)	
H2	0.1675	1.0047	0.4115	0.061*	
C3	0.2336 (3)	1.1150 (3)	0.3353 (3)	0.0528 (9)	
H3	0.2638	1.1528	0.3953	0.063*	
C4	0.2480 (3)	1.1515 (3)	0.2381 (3)	0.0506 (9)	
H4	0.2863	1.2131	0.2332	0.061*	
C5	0.2054 (3)	1.0954 (2)	0.1504 (3)	0.0398 (7)	
C6	0.1472 (2)	0.9993 (2)	0.1570 (2)	0.0365 (7)	
C7	0.2852 (3)	1.2086 (3)	0.0418 (3)	0.0536 (9)	
H7A	0.2584	1.2717	0.0683	0.080*	
H7B	0.2880	1.2184	−0.0307	0.080*	
H7C	0.3555	1.1939	0.0813	0.080*	
C8	0.1120 (2)	0.9388 (2)	0.0659 (2)	0.0367 (7)	
H8	0.1252	0.9652	0.0031	0.044*	
C9	0.0357 (2)	0.7889 (2)	−0.0328 (2)	0.0349 (7)	
C10	0.0623 (3)	0.8146 (2)	−0.1274 (2)	0.0398 (7)	
H10	0.1035	0.8743	−0.1319	0.048*	
C11	0.0285 (3)	0.7528 (3)	−0.2154 (3)	0.0417 (7)	
C12	−0.0325 (3)	0.6620 (2)	−0.2082 (2)	0.0407 (7)	
C13	−0.0573 (3)	0.6355 (2)	−0.1132 (3)	0.0421 (8)	
H13	−0.0966	0.5746	−0.1080	0.050*	
C14	−0.0241 (2)	0.6985 (2)	−0.0250 (2)	0.0347 (7)	
C15	0.0579 (3)	0.7840 (3)	−0.3177 (3)	0.0583 (10)	
H15A	0.0972	0.8491	−0.3086	0.087*	
H15B	−0.0058	0.7932	−0.3703	0.087*	
H15C	0.1010	0.7295	−0.3394	0.087*	
C16	−0.0759 (3)	0.5964 (3)	−0.3041 (3)	0.0534 (9)	
H16A	−0.1054	0.5320	−0.2833	0.080*	
H16B	−0.0194	0.5800	−0.3399	0.080*	
H16C	−0.1306	0.6353	−0.3501	0.080*	
C17	−0.1087 (3)	0.6065 (2)	0.0954 (2)	0.0405 (7)	
H17	−0.1382	0.5632	0.0393	0.049*	
C18	−0.1353 (3)	0.5845 (2)	0.1932 (3)	0.0406 (7)	
C19	−0.2085 (3)	0.5008 (3)	0.2020 (3)	0.0520 (9)	
C20	−0.2290 (3)	0.4723 (3)	0.2968 (3)	0.0583 (10)	
H20	−0.2767	0.4180	0.3016	0.070*	
C21	−0.1776 (3)	0.5255 (3)	0.3863 (3)	0.0554 (10)	
H21	−0.1906	0.5049	0.4511	0.067*	
C22	−0.1088 (3)	0.6069 (3)	0.3820 (3)	0.0514 (9)	
H22	−0.0758	0.6408	0.4435	0.062*	
C23	−0.0873 (3)	0.6399 (2)	0.2851 (3)	0.0420 (8)	
C24	−0.3272 (5)	0.3705 (4)	0.1122 (4)	0.107 (2)	
H24A	−0.2894	0.3109	0.1473	0.161*	
H24B	−0.3606	0.3507	0.0420	0.161*	
H24C	−0.3808	0.3928	0.1495	0.161*	

### Atomic displacement parameters (Å^2^)

**Table:**

	*U*^11^	*U*^22^	*U*^33^	*U*^12^	*U*^13^	*U*^23^
Ni1	0.0430 (3)	0.0344 (2)	0.0338 (2)	−0.00055 (18)	0.01401 (17)	−0.00004 (17)
O1	0.0676 (16)	0.0428 (12)	0.0370 (13)	−0.0083 (11)	0.0177 (12)	−0.0029 (10)
O2	0.0570 (15)	0.0408 (12)	0.0511 (15)	−0.0117 (11)	0.0203 (12)	−0.0070 (10)
O3	0.0599 (14)	0.0443 (12)	0.0366 (12)	−0.0058 (11)	0.0210 (11)	0.0005 (10)
O4	0.096 (2)	0.0802 (18)	0.0535 (17)	−0.0504 (17)	0.0144 (16)	0.0106 (15)
N1	0.0350 (14)	0.0370 (13)	0.0337 (14)	−0.0019 (10)	0.0117 (11)	−0.0013 (11)
N2	0.0425 (15)	0.0360 (12)	0.0342 (14)	−0.0006 (11)	0.0158 (12)	0.0034 (11)
C1	0.0447 (19)	0.0377 (16)	0.0426 (19)	0.0038 (14)	0.0134 (15)	−0.0060 (14)
C2	0.066 (2)	0.0485 (19)	0.038 (2)	0.0014 (17)	0.0135 (18)	−0.0058 (15)
C3	0.058 (2)	0.053 (2)	0.045 (2)	−0.0070 (17)	0.0061 (18)	−0.0139 (17)
C4	0.052 (2)	0.0418 (17)	0.058 (2)	−0.0081 (15)	0.0113 (18)	−0.0122 (16)
C5	0.0378 (18)	0.0377 (16)	0.046 (2)	0.0018 (13)	0.0135 (15)	−0.0065 (14)
C6	0.0364 (17)	0.0322 (14)	0.0420 (19)	0.0041 (12)	0.0108 (14)	−0.0040 (13)
C7	0.055 (2)	0.0467 (18)	0.064 (2)	−0.0115 (17)	0.0230 (19)	−0.0006 (17)
C8	0.0363 (17)	0.0362 (15)	0.0401 (18)	0.0016 (13)	0.0140 (14)	0.0005 (13)
C9	0.0370 (17)	0.0349 (14)	0.0345 (16)	−0.0018 (13)	0.0114 (13)	−0.0044 (13)
C10	0.0441 (19)	0.0398 (16)	0.0375 (18)	−0.0058 (14)	0.0129 (15)	−0.0011 (13)
C11	0.0430 (19)	0.0481 (17)	0.0375 (18)	0.0019 (14)	0.0160 (15)	−0.0002 (14)
C12	0.0450 (19)	0.0423 (17)	0.0376 (18)	−0.0009 (14)	0.0148 (15)	−0.0058 (14)
C13	0.045 (2)	0.0382 (16)	0.047 (2)	−0.0091 (14)	0.0181 (16)	−0.0056 (14)
C14	0.0340 (17)	0.0364 (15)	0.0358 (17)	−0.0010 (12)	0.0117 (13)	0.0018 (12)
C15	0.070 (2)	0.065 (2)	0.045 (2)	−0.015 (2)	0.0241 (19)	−0.0027 (18)
C16	0.059 (2)	0.059 (2)	0.045 (2)	−0.0130 (18)	0.0163 (18)	−0.0120 (17)
C17	0.0442 (19)	0.0403 (16)	0.0381 (18)	−0.0054 (14)	0.0107 (15)	0.0023 (14)
C18	0.0422 (19)	0.0401 (16)	0.0425 (19)	0.0005 (14)	0.0156 (15)	0.0092 (14)
C19	0.058 (2)	0.0480 (19)	0.052 (2)	−0.0087 (17)	0.0170 (19)	0.0094 (17)
C20	0.061 (2)	0.055 (2)	0.064 (3)	−0.0059 (18)	0.026 (2)	0.0199 (19)
C21	0.070 (3)	0.052 (2)	0.054 (2)	0.0074 (19)	0.034 (2)	0.0134 (18)
C22	0.068 (2)	0.0470 (19)	0.046 (2)	0.0061 (17)	0.0270 (19)	0.0053 (16)
C23	0.048 (2)	0.0387 (16)	0.0425 (19)	0.0098 (14)	0.0174 (16)	0.0082 (14)
C24	0.139 (5)	0.113 (4)	0.066 (3)	−0.090 (4)	0.013 (3)	0.008 (3)

### Geometric parameters (Å, °)

**Table:**

Ni1—O3	1.832 (2)	C9—C14	1.391 (4)
Ni1—O1	1.833 (2)	C10—C11	1.382 (4)
Ni1—N1	1.845 (2)	C10—H10	0.9300
Ni1—N2	1.848 (2)	C11—C12	1.402 (4)
O1—C1	1.299 (4)	C11—C15	1.509 (4)
O2—C5	1.367 (4)	C12—C13	1.380 (4)
O2—C7	1.428 (4)	C12—C16	1.509 (4)
O3—C23	1.297 (4)	C13—C14	1.392 (4)
O4—C19	1.358 (4)	C13—H13	0.9300
O4—C24	1.438 (4)	C15—H15A	0.9600
N1—C8	1.313 (4)	C15—H15B	0.9600
N1—C9	1.430 (4)	C15—H15C	0.9600
N2—C17	1.302 (4)	C16—H16A	0.9600
N2—C14	1.433 (4)	C16—H16B	0.9600
C1—C2	1.410 (4)	C16—H16C	0.9600
C1—C6	1.419 (4)	C17—C18	1.411 (4)
C2—C3	1.365 (5)	C17—H17	0.9300
C2—H2	0.9300	C18—C23	1.415 (4)
C3—C4	1.395 (5)	C18—C19	1.434 (4)
C3—H3	0.9300	C19—C20	1.363 (5)
C4—C5	1.361 (4)	C20—C21	1.390 (5)
C4—H4	0.9300	C20—H20	0.9300
C5—C6	1.437 (4)	C21—C22	1.364 (5)
C6—C8	1.407 (4)	C21—H21	0.9300
C7—H7A	0.9600	C22—C23	1.409 (4)
C7—H7B	0.9600	C22—H22	0.9300
C7—H7C	0.9600	C24—H24A	0.9600
C8—H8	0.9300	C24—H24B	0.9600
C9—C10	1.382 (4)	C24—H24C	0.9600
			
O3—Ni1—O1	83.87 (9)	C10—C11—C15	119.5 (3)
O3—Ni1—N1	177.97 (11)	C12—C11—C15	121.0 (3)
O1—Ni1—N1	94.67 (10)	C13—C12—C11	119.4 (3)
O3—Ni1—N2	94.57 (10)	C13—C12—C16	119.7 (3)
O1—Ni1—N2	178.42 (10)	C11—C12—C16	120.8 (3)
N1—Ni1—N2	86.88 (10)	C12—C13—C14	120.9 (3)
C1—O1—Ni1	128.5 (2)	C12—C13—H13	119.6
C5—O2—C7	117.1 (3)	C14—C13—H13	119.6
C23—O3—Ni1	128.4 (2)	C9—C14—C13	119.4 (3)
C19—O4—C24	116.8 (3)	C9—C14—N2	113.6 (3)
C8—N1—C9	120.8 (3)	C13—C14—N2	127.0 (3)
C8—N1—Ni1	126.2 (2)	C11—C15—H15A	109.5
C9—N1—Ni1	112.93 (18)	C11—C15—H15B	109.5
C17—N2—C14	121.3 (3)	H15A—C15—H15B	109.5
C17—N2—Ni1	125.9 (2)	C11—C15—H15C	109.5
C14—N2—Ni1	112.73 (18)	H15A—C15—H15C	109.5
O1—C1—C2	118.1 (3)	H15B—C15—H15C	109.5
O1—C1—C6	123.4 (3)	C12—C16—H16A	109.5
C2—C1—C6	118.5 (3)	C12—C16—H16B	109.5
C3—C2—C1	120.4 (3)	H16A—C16—H16B	109.5
C3—C2—H2	119.8	C12—C16—H16C	109.5
C1—C2—H2	119.8	H16A—C16—H16C	109.5
C2—C3—C4	122.2 (3)	H16B—C16—H16C	109.5
C2—C3—H3	118.9	N2—C17—C18	125.4 (3)
C4—C3—H3	118.9	N2—C17—H17	117.3
C5—C4—C3	119.0 (3)	C18—C17—H17	117.3
C5—C4—H4	120.5	C17—C18—C23	121.5 (3)
C3—C4—H4	120.5	C17—C18—C19	120.0 (3)
C4—C5—O2	124.2 (3)	C23—C18—C19	118.5 (3)
C4—C5—C6	121.0 (3)	O4—C19—C20	124.9 (3)
O2—C5—C6	114.7 (3)	O4—C19—C18	113.9 (3)
C8—C6—C1	121.7 (3)	C20—C19—C18	121.2 (3)
C8—C6—C5	119.4 (3)	C19—C20—C21	119.1 (3)
C1—C6—C5	118.8 (3)	C19—C20—H20	120.4
O2—C7—H7A	109.5	C21—C20—H20	120.4
O2—C7—H7B	109.5	C22—C21—C20	122.1 (3)
H7A—C7—H7B	109.5	C22—C21—H21	119.0
O2—C7—H7C	109.5	C20—C21—H21	119.0
H7A—C7—H7C	109.5	C21—C22—C23	120.4 (4)
H7B—C7—H7C	109.5	C21—C22—H22	119.8
N1—C8—C6	125.1 (3)	C23—C22—H22	119.8
N1—C8—H8	117.5	O3—C23—C22	118.0 (3)
C6—C8—H8	117.5	O3—C23—C18	123.3 (3)
C10—C9—C14	119.8 (3)	C22—C23—C18	118.7 (3)
C10—C9—N1	126.5 (3)	O4—C24—H24A	109.5
C14—C9—N1	113.7 (3)	O4—C24—H24B	109.5
C9—C10—C11	120.9 (3)	H24A—C24—H24B	109.5
C9—C10—H10	119.5	O4—C24—H24C	109.5
C11—C10—H10	119.5	H24A—C24—H24C	109.5
C10—C11—C12	119.5 (3)	H24B—C24—H24C	109.5
			
O3—Ni1—O1—C1	179.7 (3)	C9—C10—C11—C12	−0.8 (5)
N1—Ni1—O1—C1	1.2 (3)	C9—C10—C11—C15	179.3 (3)
O1—Ni1—O3—C23	173.2 (3)	C10—C11—C12—C13	−0.4 (5)
N2—Ni1—O3—C23	−7.1 (3)	C15—C11—C12—C13	179.5 (3)
O1—Ni1—N1—C8	−5.6 (3)	C10—C11—C12—C16	176.5 (3)
N2—Ni1—N1—C8	174.6 (3)	C15—C11—C12—C16	−3.6 (5)
O1—Ni1—N1—C9	175.77 (19)	C11—C12—C13—C14	1.1 (5)
N2—Ni1—N1—C9	−4.0 (2)	C16—C12—C13—C14	−175.8 (3)
O3—Ni1—N2—C17	8.2 (3)	C10—C9—C14—C13	−0.6 (4)
N1—Ni1—N2—C17	−173.2 (3)	N1—C9—C14—C13	178.5 (3)
O3—Ni1—N2—C14	−174.79 (19)	C10—C9—C14—N2	−179.4 (3)
N1—Ni1—N2—C14	3.8 (2)	N1—C9—C14—N2	−0.3 (4)
Ni1—O1—C1—C2	−176.2 (2)	C12—C13—C14—C9	−0.6 (5)
Ni1—O1—C1—C6	3.6 (5)	C12—C13—C14—N2	177.9 (3)
O1—C1—C2—C3	−179.7 (3)	C17—N2—C14—C9	174.4 (3)
C6—C1—C2—C3	0.4 (5)	Ni1—N2—C14—C9	−2.8 (3)
C1—C2—C3—C4	1.2 (6)	C17—N2—C14—C13	−4.3 (5)
C2—C3—C4—C5	−1.1 (5)	Ni1—N2—C14—C13	178.5 (3)
C3—C4—C5—O2	−178.6 (3)	C14—N2—C17—C18	179.8 (3)
C3—C4—C5—C6	−0.7 (5)	Ni1—N2—C17—C18	−3.4 (5)
C7—O2—C5—C4	6.2 (5)	N2—C17—C18—C23	−5.6 (5)
C7—O2—C5—C6	−171.8 (3)	N2—C17—C18—C19	177.4 (3)
O1—C1—C6—C8	−5.1 (5)	C24—O4—C19—C20	−1.2 (6)
C2—C1—C6—C8	174.8 (3)	C24—O4—C19—C18	179.3 (4)
O1—C1—C6—C5	178.1 (3)	C17—C18—C19—O4	−5.8 (5)
C2—C1—C6—C5	−2.1 (4)	C23—C18—C19—O4	177.2 (3)
C4—C5—C6—C8	−174.6 (3)	C17—C18—C19—C20	174.7 (3)
O2—C5—C6—C8	3.4 (4)	C23—C18—C19—C20	−2.3 (5)
C4—C5—C6—C1	2.3 (5)	O4—C19—C20—C21	−179.5 (4)
O2—C5—C6—C1	−179.7 (3)	C18—C19—C20—C21	−0.1 (6)
C9—N1—C8—C6	−175.9 (3)	C19—C20—C21—C22	1.3 (6)
Ni1—N1—C8—C6	5.6 (4)	C20—C21—C22—C23	−0.1 (5)
C1—C6—C8—N1	0.3 (5)	Ni1—O3—C23—C22	−178.7 (2)
C5—C6—C8—N1	177.1 (3)	Ni1—O3—C23—C18	0.8 (4)
C8—N1—C9—C10	3.6 (5)	C21—C22—C23—O3	177.2 (3)
Ni1—N1—C9—C10	−177.7 (3)	C21—C22—C23—C18	−2.4 (5)
C8—N1—C9—C14	−175.4 (3)	C17—C18—C23—O3	7.0 (5)
Ni1—N1—C9—C14	3.3 (3)	C19—C18—C23—O3	−176.0 (3)
C14—C9—C10—C11	1.3 (5)	C17—C18—C23—C22	−173.5 (3)
N1—C9—C10—C11	−177.6 (3)	C19—C18—C23—C22	3.5 (5)

### Hydrogen-bond geometry (Å, °)

**Table:**

*D*—H···*A*	*D*—H	H···*A*	*D*···*A*	*D*—H···*A*
C7—H7C···O3^i^	0.96	2.51	3.424 (5)	158
C21—H21···O2^ii^	0.93	2.52	3.340 (4)	147

Symmetry codes: (i) −*x*+1/2, *y*+1/2, −*z*+1/2; (ii) *x*−1/2, −*y*+3/2, *z*+1/2.
